# Synthesis of Intermetallic (Mg_1−x_,Al_x_)_2_Ca by Combinatorial Sputtering

**DOI:** 10.3390/ma12183026

**Published:** 2019-09-18

**Authors:** Philipp Keuter, Soheil Karimi Aghda, Denis Music, Pauline Kümmerl, Jochen M. Schneider

**Affiliations:** Materials Chemistry, RWTH Aachen University, Kopernikusstr. 10, 52074 Aachen, Germany; karimi@mch.rwth-aachen.de (S.K.A.); music@mch.rwth-aachen.de (D.M.); schneider@mch.rwth-aachen.de (J.M.S.)

**Keywords:** physical vapor deposition, desorption, combinatorial sputtering, intermetallics, magnesium, density functional theory

## Abstract

The synthesis–composition–structure relationship in the Mg–Ca–Al system is studied using combinatorial magnetron sputtering. With increasing deposition temperature, a drastic decrease in Mg concentration is obtained. This behavior can be understood based on density functional theory calculations yielding a desorption energy of 1.9 eV/atom for Mg from a hexagonal Mg nanocluster which is far below the desorption energy of Mg from a Mg_2_Ca nanocluster (3.4 eV/atom) implying desorption of excess Mg during thin film growth at elevated temperatures. Correlative structural and chemical analysis of binary Mg–Ca thin films suggests the formation of hexagonal Mg_2_Ca (C14 Laves phase) in a wide Mg/Ca range from 1.7 to 2.2, expanding the to date reported stoichiometry range. Pronounced thermally-induced desorption of Mg is utilized to synthesize stoichiometric (Mg_1−x_,Al_x_)_2_Ca thin films by additional co-sputtering of elemental Al, exhibiting a higher desorption energy (6.7 eV/atom) compared to Mg (3.4 eV/atom) from Mg_2_Ca, which governs its preferred incorporation during synthesis. X-ray diffraction investigations along the chemical gradient suggest the formation of intermetallic C14 (Mg_1–x_,Al_x_)_2_Ca with a critical aluminum concentration of up to 23 at.%. The introduced synthesis strategy, based on the thermally-induced desorption of weakly bonded species, and the preferential incorporation of strongly bonded species, may also be useful for solubility studies of other phases within this ternary system as well as for other intermetallics with weakly bonded alloying constituents.

## 1. Introduction

Hexagonal close-packed magnesium (Mg) combines promising properties for structural applications considering that it exhibits one of the lowest densities among metals (1.7 g/cm³) as well as a high specific strength, high abundance, and good castability [1,2]. The main alloying element for commercial Mg alloys is aluminum (Al), promoting corrosion resistance [3]. Nevertheless, most Mg–Al alloys undergo a rapid degradation of creep resistance at elevated temperatures restricting their implementation in industry to non-critical parts [1,4,5]. However, the addition of calcium (Ca) can enhance the creep resistance, which is commonly reasoned by the precipitation of intermetallic (Mg,Al)_2_Ca Laves phases [4,5,6,7,8].

Within the ternary Mg–Al–Ca system, the cubic (C15) Al_2_Ca and the hexagonal (C14) Mg_2_Ca phases are reported to be the thermodynamically stable binary Laves phases with an extensive solubility of the third constituent, Mg in Al_2_Ca and Al in Mg_2_Ca [9]. Recent experiments and first-principle calculations also suggest the existence of a dihexagonal C36 (Mg,Al)_2_Ca phase [9,10,11,12]. Despite the numerous theoretical and experimental studies, a systematic investigation, especially of the ternary compounds, is missing due to limited composition-spreads that can efficiently be covered using conventional bulk synthesis approaches. Furthermore, contradictive information about the stoichiometry range of binary Mg_2_Ca [13,14] and the critical Al solubility in C14 Mg_2_Ca, ranging from 16 to 25 at.% [11,13,14], are reported in the literature. 

Combinatorial magnetron sputtering, as an efficient material synthesis approach, in combination with high-throughput spatially-resolved chemical composition and crystal structural analysis is ideally suited to systematically study the composition-induced phase formation in complex systems [15]. However, desorption of volatile film-forming species during sputtering at elevated temperatures is a well-known phenomenon making sputtering of Mg–Ca–Al challenging. For example, desorption of Mg and Al has been observed at 600 °C for magnetron sputtered Al–Mg–B thin films [16]. Furthermore, evaporation of Al above 700 °C has been reported upon sputtering from a Ti_2_AlC compound target [17]. 

To address the open questions regarding the stoichiometry range of binary Mg_2_Ca and the critical Al solubility in C14 Mg_2_Ca, a high-throughput approach was employed. We carried out a systematic exploration of the synthesis temperature-dependent composition and resulting structural evolution of sputtered binary Mg–Ca thin films. A non-linear deposition temperature dependency of the Mg/Ca ratio was obtained which was subsequently studied theoretically, considering competitive surface processes including adatom incorporation and desorption of weakly bonded species. The combination of preferred Al incorporation and desorption of excess Mg at elevated temperatures was utilized to synthesize stoichiometric (Mg,Al)_2_Ca thin films by co-sputtering of elemental Al. Furthermore, the composition-induced phase formation of C14 (Mg,Al)_2_Ca was systematically investigated. 

## 2. Materials and Methods

Thin film depositions were carried out by magnetron sputtering in a laboratory-scale high vacuum chamber evacuated to a base pressure below 1 × 10^−4^ Pa. All depositions were conducted at a constant argon partial pressure of 0.5 Pa. The utilized Si (100) substrates were positioned at a target-to-substrate distance of 10 cm. The set temperature, corresponding to the substrate temperature prior to the ignition of the plasma, was varied between room temperature (no intentional heating) and 300 °C. A circular Mg-Ca target (50 mm in diameter) was produced from a purchased Mg–Mg_2_Ca composite material with a composition of Mg83–Ca17 at.% determined by energy dispersive X-ray analysis (EDX). The target was operated in pulsed direct-current mode with an ENI RPG-100E generator at a frequency of 250 kHz and duty cycle of 60%. The target was powered with 60 and 200 W for the deposition of binary Mg–Ca and ternary Mg–Ca–Al samples, respectively. The Al target (99.99% purity; 50 mm in diameter) power was varied from 20 to 100 W. While the composite target was facing the substrate, the Al target was mounted at an inclination angle of 45° relative to the substrate normal resulting in a compositional gradient (see below).

Chemical composition analysis of the combinatorial Mg–Ca–Al films was conducted by EDX utilizing a JEOL JSM-6480 scanning electron microscope (JEOL Ltd., Tokyo, Japan) equipped with an EDAX Genesis 2000 analyzer (EDAX Inc., Mahwah, NJ, USA). An acceleration voltage of 8 kV was used. Spatially-resolved structural analysis was conducted by X-ray diffraction (XRD) using a Bruker AXS D8 Discover (Bruker Corporation, Billerica, MA, USA) General Area Detector Diffraction System (GADDS) with a pinhole-collimated primary beam of 0.5 mm. The incidence angle ω was fixed to 15° whereas the 2θ angle was increased from 15° to 75° during the measurements. The voltage and current of the Cu Kα radiation sources were set to 40 kV and 40 mA, respectively. For the structural identification of hexagonal Mg, the JCPDS card no. 35–0821 was used.

To estimate desorption energies of Mg, Ca, and Al adatoms from Mg and Mg_2_Ca nanoclusters, mimicking fundamental surface processes during the growth of Mg-Ca thin films with Al additions, density functional theory (DFT) [18] at 0 K was employed. A linear combination of localized quasiatomic orbitals [19] and the generalized gradient approximation was used [20] within the OpenMX software package [21]. The basis functions were defined employing a confinement scheme [19,22] and described as: Mg7.0-s3p3d2, Ca9.0-s4p3d2, and Al7.0-s3p3d2 (the leading symbol denotes the chemical element, followed by the cut-off radius in the Bohr radii, and the last symbol designates the primitive orbitals). An energy cut-off of 150 Ry was chosen to reach a total energy convergence of 10^−6^ Ry. No periodic boundary conditions were applied. The desorption energy, defined herein as positive, was calculated by evaluating the total energy difference between the nanocluster + adatom (an adatom at a bulk equilibrium position) and the isolated nanocluster + isolated adatom. A Mg nanocluster was constructed by adopting an isostructural (hexagonal close-packed) Ti nanocluster containing 13 atoms [23]. A Mg terminated Mg_2_Ca nanocluster was formed based on an isostructural hexagonal C14 Fe_2_W nanocluster comprised of 125 atoms [24].

## 3. Results and Discussion

### 3.1. Binary Mg–Ca

**Figure 1:**
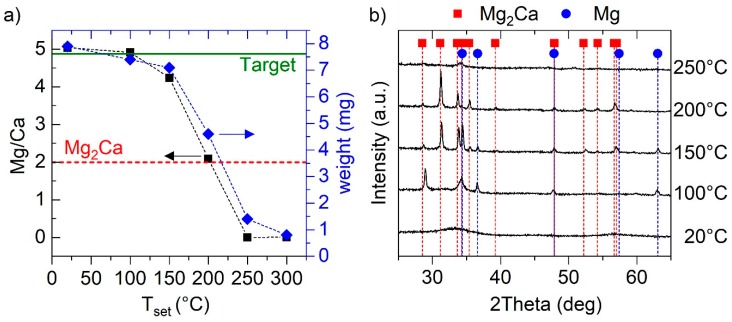
(**a**) Measured Mg/Ca ratio (EDX) and weight of as-deposited Mg–Ca thin films as a function of the set temperature. The temperature was varied from room temperature (no intentional heating, approximately 20 °C) to 300 °C, while keeping the deposition time (60 min) constant. As a guide for the eye, the Mg/Ca ratios of the utilized sputtering target (horizontal solid line) and of stoichiometric intermetallic Mg_2_Ca (horizontal dashed line) are added. (**b**) Corresponding diffractograms of the samples deposited at the indicated temperatures.

First, the synthesis–composition–structure relationship is studied for binary Mg–Ca. Depositions were conducted at varying set temperatures between room temperature and 300 °C. The Mg–Ca thin films are characterized regarding their chemical composition (Figure 1a) and crystal structure (Figure 1b).

For the samples deposited without intentional heating and 100 °C, the measured Mg/Ca ratios match the composition of the utilized composite Mg–Mg_2_Ca sputtering target. With a further increase in set temperature, the Mg/Ca ratio exhibits a drastic decrease with no Mg detectable in the films synthesized at temperatures ≥250 °C. Consequently, within the temperature range from 150 to 250 °C the chemical composition is extremely sensitive to minute temperature changes implying thermally-induced desorption of Mg during thin film growth as the species with the higher vapor pressure compared to Ca [25]. Furthermore, the weight of the thin films was determined by measuring the weight of the substrate prior to and after the deposition. The functional dependence of the thin film weight on temperature agrees well with the measured trend in Mg/Ca ratio supporting the notion of Mg desorption. Due to partial desorption of Mg, the sample synthesized at 200 °C exhibits the stoichiometry of intermetallic Mg_2_Ca. XRD analysis revealed an amorphous or nanocrystalline structure of the sample synthesized at room temperature. Upon a temperature increase to 100 and 150 °C, representative peaks for hexagonal Mg at 37° and 63° as well as for Mg_2_Ca [26] at 29°, 31°, 34° and 35.5° are obtained indicating the presence of a Mg–Mg_2_Ca phase mixture, being in agreement with chemical composition data. It may be speculated that the peak shift of 0.5° for the Mg_2_Ca diffraction peak at 29° towards higher diffraction angles [26] originates from an off-stoichiometric Laves phase composition. Since the sample is enriched in Mg compared to stoichiometric Mg_2_Ca, some Ca atoms may be replaced by smaller Mg atoms, hence, leading to a decrease in lattice spacing as observed by the shifts to higher *2*θ values. Off-stoichiometric formation of C14 Mg_2_Ca will further be discussed below. For the film deposited at 200 °C, no characteristic Mg peaks are obtained any longer, but all acquired diffraction peaks are attributed to intermetallic Mg_2_Ca in accordance with the measured composition. Hence, XRD phase pure C14 Mg_2_Ca is obtained at 200 °C, despite the usage of a Mg enriched target, due to desorption of excess Mg. However, at set temperatures above 200 °C, no Mg remains in the growing film as indicated by both, the chemical and structural analysis. 

**Figure 2:**
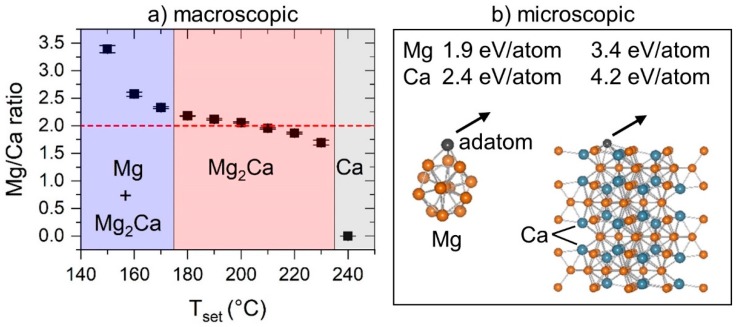
(**a**) Measured Mg/Ca ratio (EDX) of the as-deposited Mg–Ca thin films as a function of the set temperature between 150 and 240 °C. Obtained error bars lie within the data points. The horizontal dashed line indicates the stoichiometry of Mg_2_Ca. The temperature ranges are highlighted according to the obtained phases by X-ray diffraction. (**b**) Desorption energies of Mg and Ca (adatoms) from Mg (left) and from Mg_2_Ca (right) nanoclusters are calculated. Arrows indicate the desorbing Mg and Ca adatoms.

As a next step, the set temperature range between 150 and 250 °C, identified as critical for the resulting thin film composition and, hence, structure evolution, was studied in more detail. For this purpose, the set temperature was varied in steps of 10 °C between the individual depositions. The results of the experimental and theoretical study are illustrated in Figure 2. 

In the investigated temperature range in Figure 2, the Mg/Ca ratio shows a trend comparable to a tangent function, separating two temperature regions in which a drastic decrease in Mg/Ca is obtained. XRD revealed a phase mixture of Mg and Mg_2_Ca for the thin films synthesized at 150, 160, and 170 °C. Between 180 and 230 °C, the Mg/Ca ratio gradually but slightly decreases from 2.2 to 1.7, which corresponds to Mg concentrations of 68.5 and 63 at.%, respectively. Hence, the results suggest off-stoichiometric formation of intermetallic Mg_2_Ca in the given composition range, extending the range reported by Suzuki et al., who found a solubility of excess Mg in Mg_2_Ca of 4.5 at.% using bulk samples [13]. In contrast, Kevorkov et al. [14] reported Mg_2_Ca to be a line compound based on a diffusion couple study. At 240 °C, all sputtered Mg is desorbed from the surface resulting in the formation of fcc Ca. 

The obtained non-linear temperature dependency of the Mg/Ca ratio, following the trend of a tangent function, clearly cannot be explained based on Mg vapor pressure considerations alone. To mimic surface processes active during the growth of Mg–Ca thin films, desorption energies of a Mg adatom on hexagonal close-packed Mg and a hexagonal C14 Mg_2_Ca nanocluster were calculated using DFT, yielding 1.9 and 3.4 eV/atom, respectively. These values are lower than the corresponding predicted desorption energies for Ca of 2.4 and 4.2 eV/atom, respectively. For comparison, required desorption energies for K and Na from Al (001) and Al (111) are reported to lie in the range between 1.1 and 1.8 eV/atom [27]. The energetically favored Mg desorption compared to the desorption of Ca for both considered configurations is in agreement with the experimentally observed Mg loss. Furthermore, Mg desorption from a Mg nanocluster is energetically favored compared to the Mg desorption from a Mg_2_Ca nanocluster, and is, hence, suggested as the governing mechanism for set temperatures of up to ~170 °C. In contrast, the adatom Mg on Mg_2_Ca, due to the stronger bond formation between Mg and Ca, is not desorbing and readily incorporated into the growing film. As a consequence, in the temperature range of ~180 to 230 °C, the formation of intermetallic Mg_2_Ca is observed, in which all weakly bonded excess Mg is desorbed. At a set temperature of 240 °C, the formation of fcc Ca is observed indicating the desorption of all sputtered Mg from the thin film surface. Rapid degradation of the crystal quality of fcc Ca upon exposure to atmosphere, due to its high reactivity with oxygen, is observed (see absence of crystalline peaks in Figure 1b at a set temperature of 250 °C).

### 3.2. Ternary Mg–Ca–Al

**Figure 3:**
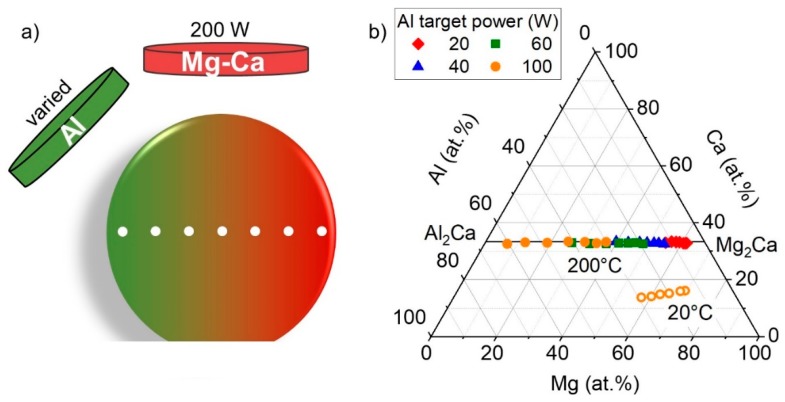
(**a**) Schematic representation of the deposition setup for combinatorial Mg–Ca–Al thin film deposition with varying Al target power. (**b**) Measured chemical composition by EDX along the chemical gradient for samples synthesized at room temperature (approximately 20 °C) and 200 °C.

Based on the data for sputtered Mg–Ca thin films, desorption of excess Mg has been identified as a beneficial mechanism for the synthesis of stoichiometric intermetallic Mg_2_Ca. To study the synthesis–composition–structure relationship along the quasi-binary Mg_2_Ca–Al_2_Ca, stoichiometric (Mg,Al)_2_Ca samples are required. Here, we seek to exploit the above identified desorption mechanism in Mg_2_Ca to grow stoichiometric (Mg,Al)_2_Ca thin films. For this purpose, the desorption energy of Al from a Mg_2_Ca nanocluster is predicted using DFT yielding 6.7 eV/atom, which is compared to the desorption energy of Mg of 3.4 eV/atom. Hence, the energetic barrier for Al desorption is considerably higher than that for Mg, indicating the formation of stronger bonds between an Al adatom and underlying intermetallic Mg_2_Ca compared to a Mg adatom. In comparison, the desorption energy of Al on a Mg_2_Ca nanocluster lies in the range of O desorption from fcc Al(111), which has been reported to require 7.3 eV/atom [28]. Consequently, our calculations suggest that Al is preferably incorporated into the intermetallic (Mg,Al)_2_Ca phases. By choosing a set temperature above the desorption temperature of excess Mg, e.g., 200 °C (see Section 3.1), we seek to exploit the above identified preferential desorption of excess Mg to grow stoichiometric (Mg,Al)_2_Ca thin films along the Mg_2_Ca–Al_2_Ca gradient. In order to critically appraise this hypothesis, Mg–Al–Ca thin films were deposited by co-sputtering of Al with varying DC target power values as well as set temperatures. The deposition setup and the resulting gradient in composition is schematically illustrated in Figure 3a. The obtained thin film composition, measured along the gradient for the various samples by EDX, is depicted in Figure 3b.

**Figure 4:**
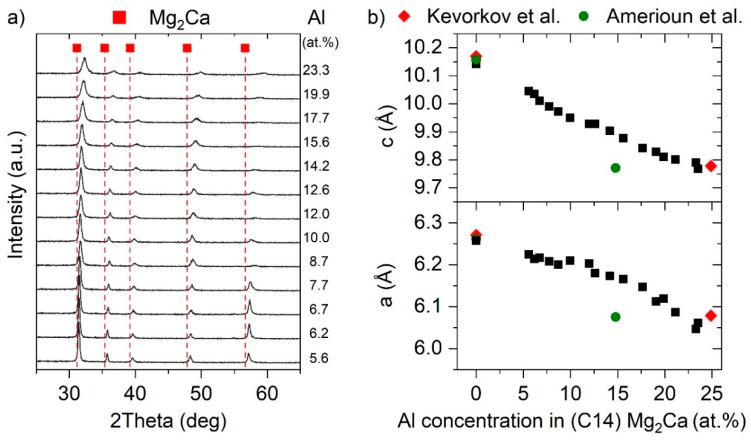
(**a**) Diffractograms of the Mg-rich ternary thin films with varying Al concentration deposited at 200 °C. (**b**) Calculated lattice parameters *a* and *c* of hexagonal C14 Mg_2_Ca as a function of the Al concentration. Experimental values from literature are added for comparison (Kevorkov et al. [14]; Amerioun et al. [11]).

At room temperature (approximately 20 °C), a chemical gradient for all sputtered species, i.e., Al, Mg, and Ca, was obtained (see Figure 3b), which can be expected based on the employed deposition setup (see Figure 3a). Furthermore, a constant Mg/Ca ratio was measured along the Al gradient, proving that Mg is not desorbing, as anticipated for such a low temperature. Upon an increase in set temperature to 200 °C, the gradient in Ca vanished and the Mg concentration was significantly reduced. As a result, the measured thin film compositions lie on the quasi-binary of Mg_2_Ca and Al_2_Ca with variations in the measured Ca concentration of less than 0.5 at.%. With increasing Al target power, the measured Al concentration increased solely at the expense of Mg resulting in thin film stoichiometries of (Mg_1−x_,Al_x_)_2_Ca with x ranging from 0 to 0.9. These results verify the hypothesis, derived based on the DFT predictions, that the sputtered Al replaces the Mg in (Mg,Al)_2_Ca, subsequently triggering its desorption as observed already for binary Mg–Ca. Thus, a synthesis strategy for stoichiometric Mg–Ca–Al thin films along the quasi-binary Mg_2_Ca–Al_2_Ca is proposed and validated, enabling the efficient study of the composition-induced phase formation. The results are presented in Figure 4. 

XRD analysis revealed that all obtained peaks can be attributed to the hexagonal C14 structure of Mg_2_Ca [26] for Al concentrations between 5.6 and 23.3 at.%. With an increase in Al concentration, a continuous peak shift towards larger 2θ angles is obtained, indicating Al incorporation into the C14 structure at least up to 23.3 at.%. These data are in agreement with bulk studies, reporting an Al solubility of 22 [13] and 25 at.% [14], respectively, supporting that Mg_2_Ca exhibits a significantly higher critical Al solubility than 16 at.%, as claimed by Amerioun et al. [11]. With increasing Al concentration from 5.6 to 23.3 at.%, an increase in full width at half maximum by a factor of 2.7 is obtained, evaluated based on the (103) peak. The peak broadening at even higher Al concentrations (not shown), eventually indicating a decreased crystal quality or competitive phase formation, impedes a meaningful discussion of the XRD data at higher alloying concentrations. 

By employing Bragg’s law [29], the lattice parameters *a* and *c* can be calculated based on the XRD data. The results are depicted in Figure 4b. The lattice parameters of binary Mg_2_Ca synthesized at 200 °C are also added. With increasing Al concentrations solved in hexagonal C14 Mg_2_Ca, a continuous decrease in lattice parameters *a* and *c* is obtained, which can be reasoned by the smaller atomic radius of Al (1.25 Å) compared to Mg (1.4 Å) [30]. Calculation of the hypothetical C14 Al_2_Ca phase yields lattice parameters *a* and *c* of 5.67 and 9.23 Å [31], respectively, which are consistent with the composition induced change in lattice parameters measured here. The obtained trend is also consistent with bulk studies, showing a good quantitative agreement with Kevorkov et al. [14], while the decrease in lattice parameters reported by Amerioun et al. [11] appears to be overestimated. It should be noted that compositional gaps in the composition–structure relationship known for the bulk samples are now filled employing the combinatorial thin film methodology. Considering the high reactivity of Ca, hampering experimental investigations, the pioneered usage of less-reactive intermetallic Mg_2_Ca as a source for Ca, presented within this work, serves as a sophisticated approach to systematically study the Mg–Ca–Al system.

## 4. Conclusions

Combinatorial magnetron sputtering from a composite Mg_2_Ca–Mg (Mg83–Ca17 at.%) and an elemental Al target was utilized to synthesize Mg–Ca–Al thin films. The obtained Mg concentration was found to be extremely sensitive to minute changes in set temperature (substrate temperature prior to plasma ignition). To mimic processes active on the thin film surface during synthesis, desorption energies of the individual species from a hexagonal close-packed Mg and a hexagonal C14 Mg_2_Ca nanocluster were calculated using DFT. The calculations suggest a desorption energy of 1.9 eV/atom for Mg from a hexagonal Mg nanocluster which is significantly lower than the desorption energy of Mg from a Mg_2_Ca nanocluster (3.4 eV/atom), implying temperature-induced desorption of excess Mg during thin film growth at elevated temperatures. Consequently, XRD phase pure Mg_2_Ca was synthesized at set temperatures between 180 and 230 °C due to desorption of excessively sputtered Mg that cannot be incorporated in Mg_2_Ca. Correlative structural and chemical analysis suggests the off-stoichiometric formation of Mg_2_Ca in a Mg/Ca ratio range from 1.7 to 2.2. Above 240 °C, the formation of fcc Ca indicates desorption of all sputtered Mg from the thin film surface. Furthermore, the desorption energy of Al from Mg_2_Ca was calculated to be 6.7 eV/atom, which is almost twice the desorption energy of Mg from a Mg_2_Ca nanocluster, implying a preferred incorporation of Al instead of Mg in (Mg,Al)_2_Ca during sputtering. Consequently, this mechanism of excess Mg evaporation at elevated synthesis temperatures was exploited experimentally by co-sputtering of Al, resulting in the formation of stoichiometric chemically-graded (Mg_1−x_,Al_x_)_2_Ca with *x* varying from 0 to 0.9. Systematic investigation of the composition–structure relationship along the chemical gradient suggests the formation of hexagonal C14 Mg_2_Ca with a significantly higher critical aluminum concentration as reported by Amerioun et al. [11], in agreement with Suzuki et al. [13] and Kevorkov et al. [14]. The introduced synthesis strategy, based on the thermally-induced desorption of weakly bonded species, and the preferential incorporation of strongly bonded species, enabled the systematic investigation of the critical Al solubility of the ternary (Mg_1−x_,Al_x_)_2_Ca intermetallic phase. This approach may also be useful for solubility studies of other phases within this ternary system as well as for other intermetallics exhibiting weakly and stongly bonded alloying constituents.
